# The pre- and postoperative nomograms to predict the textbook outcomes of patients who underwent hepatectomy for hepatocellular carcinoma

**DOI:** 10.3389/fonc.2023.1089716

**Published:** 2023-04-14

**Authors:** Gui-Lin Xie, Lei Liang, Tai-Wei Ye, Fei-Qi Xu, Dong-Dong Wang, Ya-Ming Xie, Kang-Jun Zhang, Tian-Wei Fu, Wei-Feng Yao, Jun-Wei Liu, Cheng-Wu Zhang

**Affiliations:** ^1^ Department of Hepatobiliary Surgery, Affiliated Hospital of Shaoxing University, Shaoxing, Zhejiang, China; ^2^ General Surgery, Cancer Center, Department of Hepatobiliary and Pancreatic Surgery and Minimally Invasive Surgery, Zhejiang Provincial People’s Hospital, Affiliated People’s Hospital, Hangzhou Medical College, Hangzhou, Zhejiang, China; ^3^ The Second School of Clinical Medicine, Zhejiang Chinese Medical University, Hangzhou, China

**Keywords:** hepatocellular carcinoma, textbook outcomes, nomogram, hepatectomy, laparoscopic

## Abstract

**Background and aims:**

An increasing number of studies have confirmed that non-textbook outcomes (non-TO) are a risk factor for the long-term outcome of malignant tumors. It is particularly important to identify the predictive factors of non-TO to improve the quality of surgical treatment. We attempted to construct two nomograms for preoperative and postoperative prediction of non-TO after laparoscopic hepatectomy for hepatocellular carcinoma (HCC).

**Methods:**

Patients who underwent curative-intent hepatectomy for HCC between 2014 and 2021 at two Chinese hospitals were analyzed. Using univariate and multivariate analyses, the independent predictors of non-TO were identified. The prediction accuracy is accurately measured by the receiver operating characteristic (ROC) curve and calibration curve. ROC curves for the preoperative and postoperative models, Child–Pugh grade, BCLC staging, and 8th TNM staging were compared relative to predictive accuracy for non-TO.

**Results:**

Among 515 patients, 286 patients (55.5%) did not achieve TO in the entire cohort. Seven and eight independent risk factors were included in the preoperative and postoperative predictive models by multivariate logistic regression analysis, respectively. The areas under the ROC curves for the postoperative and preoperative models, Child–Pugh grade, BCLC staging, and 8th TNM staging in predicting non-TO were 0.762, 0.698, 0.579, 0.569, and 0.567, respectively.

**Conclusion:**

Our proposed preoperative and postoperative nomogram models were able to identify patients at high risk of non-TO following laparoscopic resection of HCC, which may guide clinicians to make individualized surgical decisions, improve postoperative survival, and plan adjuvant therapy against recurrence.

## Introduction

Primary liver cancer is the seventh most common cancer disease and is the second leading cause of cancer-related death (1). Hepatocellular carcinoma (HCC) is still the most common form of primary liver cancer, accounting for 90% of medical records (2). Clinically adopted curative treatment methods for HCC include open or minimally invasive liver resection, radiofrequency ablation, and liver transplantation. Transcatheter arterial chemoembolization (TACE) and targeted therapy and immunotherapy are used as adjuvant or neoadjuvant therapy for HCC patients. While multimodal treatment is well known to gain a significant impact on the prognosis of patients with HCC, the outcomes are still far from satisfactory. Thus, it is critical to investigate which clinical factors are associated with improved overall survival (OS) in patients with HCC.

Previous studies had shown that intraoperative blood transfusion (3–5) as well as postoperative complications (6, 7) representing the perioperative medical quality have a far-reaching influence on the OS for HCC patients. Nevertheless, for patients with HCC who need surgical treatment, it is not enough to use a single variable to assess the impacts on different individuals. “Textbook outcomes”, as a comprehensive indicator, have been reported extensively, evaluating surgical quality and safety. Many studies have previously demonstrated patients with malignancies, such as esophageal cancer (8–11), colon cancer (12), lung cancer (13), primary liver cancer (14, 15), and soft tissue sarcoma (16), who achieved TO, representing the ideal clinical procedure, which could improve long-term outcomes.

Compared with open liver resection, laparoscopic liver resection (LLR) tends to reach TO, which reflects the advantages of minimally invasive surgery (17).

LLR of anterolateral segments of liver was considered as a standard operation and the relationship between LLR of anterolateral hepatic segments with TO had been explored in a previous study (15). However, with the increasing maturity of LLR technology, the use of minimally invasive surgical approach in other segments has also been widely performed. Therefore, it is crucial to comprehensively analyze which factors affect the TO of LLR. The aim of the present study is to identify the predictors of non-TO, and implement corresponding preoperative intervention for patients who would undergo LLR. In addition, using a multicenter database, preoperative and postoperative nomogram models were conducted to predict non-TO.

## Patients and methods

### Patients and study design

Consecutive patients with HCC who received curative-intention LLR in Zhejiang Provincial People’s Hospital and Shaoxing Municipal Hospital from 2016 to December 2021 were enrolled. Exclusion criteria include the following: (1) repeat liver resection for recurrent HCC, (2) under 18 years of age, (3) traditional open hepatectomy, and (4) important dates or data missing related to TO. HCC patients were initially differentiated according to dynamic CT or MRI. If the imaging diagnostic characteristics in CT or MR are special for HCC (strong contrast medium intake in arterial phase, and extracellular contrast medium flushing out in venous phase and/or delayed phase), then all HCC will be diagnosed by pathology of patient samples. This retrospective study was in line with the Helsinki Declaration and was approved by the Institutional Ethics Committee, and the need for informed consent was abandoned.

### Clinicopathological variables

The preoperative, intraoperative, and postoperative clinical variables were prospectively and retrospectively collected from the medical record system of Zhejiang Provincial People’s Hospital and Shaoxing Municipal Hospital. Preoperative variables included age at surgery; sex; body mass index (BMI); American Society of Anesthesiologists (ASA) score; history of alcohol drinking, diabetes mellitus, and cigarette smoking; hepatitis B virus (HBV); presence of cirrhosis and portal hypertension; Child–Pugh grade; preoperative levels of alpha-fetoprotein (AFP); albumin–bilirubin (ALBI) score; neutrophil-to-lymphocyte ratio (NLR) score; alanine aminotransferase (ALT); aspartate transaminase (AST); preoperative platelet count; maximum diameter of tumor; tumor location; tumor number; and macrovascular invasion through preoperative imaging. Intraoperative variables included intraoperative blood loss, type of resection, and extent of hepatectomy. In this study, obesity was defined as BMI ≥ 28 kg/m^2^. According to the ALBI score classification: ALBI score ≤ −2.6 (grade I), −2.6 < ALBI score ≤ −1.39 (grade II), and ALBI score > −1.39 (grade III). High ALBI grade was defined as having ALBI grade II/III, and normal ALBI grade was defined as having an ALBI score ≤ −2.6 (grade I) (18). The NLR score divided patients into two groups: score ≤ 2.81 (low grade) and score > 2.81 (high grade) (19). Tumor number ≥ 2 was defined as multiple tumors. The extent of hepatectomy was divided into major or minor liver resection. Hepatectomy was classified as anatomical and non-anatomical based on Brisbane 2000 criteria (20). All the serum samples were collected in the morning when the patient had not eaten for more than 8 h. The information was obtained before all the treatments and less than 1 week before the operation. All independent variables of serological tests were tested by clinical laboratories of two hospitals.

### Textbook outcome

In the present study, TO consists of six parameters, namely, (1) without 30-day morbidity after surgery; (2) no prolonged duration of hospital stays; (3) no perioperative blood transfusion; (4) no readmission within 30 days after discharge (21); (5) without 90-day mortality after surgery; and (6) R0 resection. Postoperative morbidities include liver failure, bile leakage or other biliary complications, hemorrhage, infection from a variety of causes, and cardiovascular, brain, pulmonary, renal, and other complications. According to the criterion for the prolonged length of hospital stay after surgery (17), we defined 10 days as the cutoff value. The negative result of both microscopic and macroscopic observations of resection margin was defined as R0 resection (22). If the above six conditions were met, TO of LLR was considered achieved; otherwise, it is non-TO.

### Definition of Child–Pugh grade, BCLC staging, and 8th TNM staging

The Child–Pugh grade was defined as follows: grade A (5–6 points), grade B (7–9 points), and grade C (10–15 points). In this study, there were no patients with Child–Pugh grade C. BCLC staging was classified as very early stage (BCLC 0), early stage (BCLC-A), intermediate stage (BCLC-B), advanced stage (BCLC-C), and end-stage (BCLC-D) based on tumor burden, liver function, and performance status. We defined BCLC 0/A as early stage and there were no patients with BCLC-D in our study. The 8th TNM staging system is mainly based on factors associated with tumor size and number, vascular invasion, invasion of visceral peritoneum, and lymph node or distant metastasis.

### Statistical analysis

The statistical analysis was carried out using the SPSS 25.0 (SPSS, Inc) and R 4.2.1 (http://www.r-project.org/). The categorical variables are indicated by number (*n*) and percentage (%). Comparison of categorical variables shall be adopted as appropriate *χ* ^2^ test or Fisher exact test. Univariate and multivariate logistic regression analysis was performed to determine independent preoperative predictors of non-TO. In univariate analysis, the variables with *p* < 0.1 were entered into the multivariate regression model using the forward stepwise variable selection method. Two nomograms were built up on the basis of the results of the multivariate analysis of the preoperative data. The nomogram was subjected to 1,000 bootstrap resamples for internal validation of each cohort. The model performance for predicting outcome was evaluated by calculating the area under the receiver operating characteristic curve (AUC) (23). Evaluate the calibration of the nomogram according to the calibration curve. The results predicted by the accurate measurement model of the calibration curve are related to the conclusions seen in the queue. *p* < 0.05 was considered statistically significant.

## Results

### Comparisons of TO and non-TO

**Figure 1:**
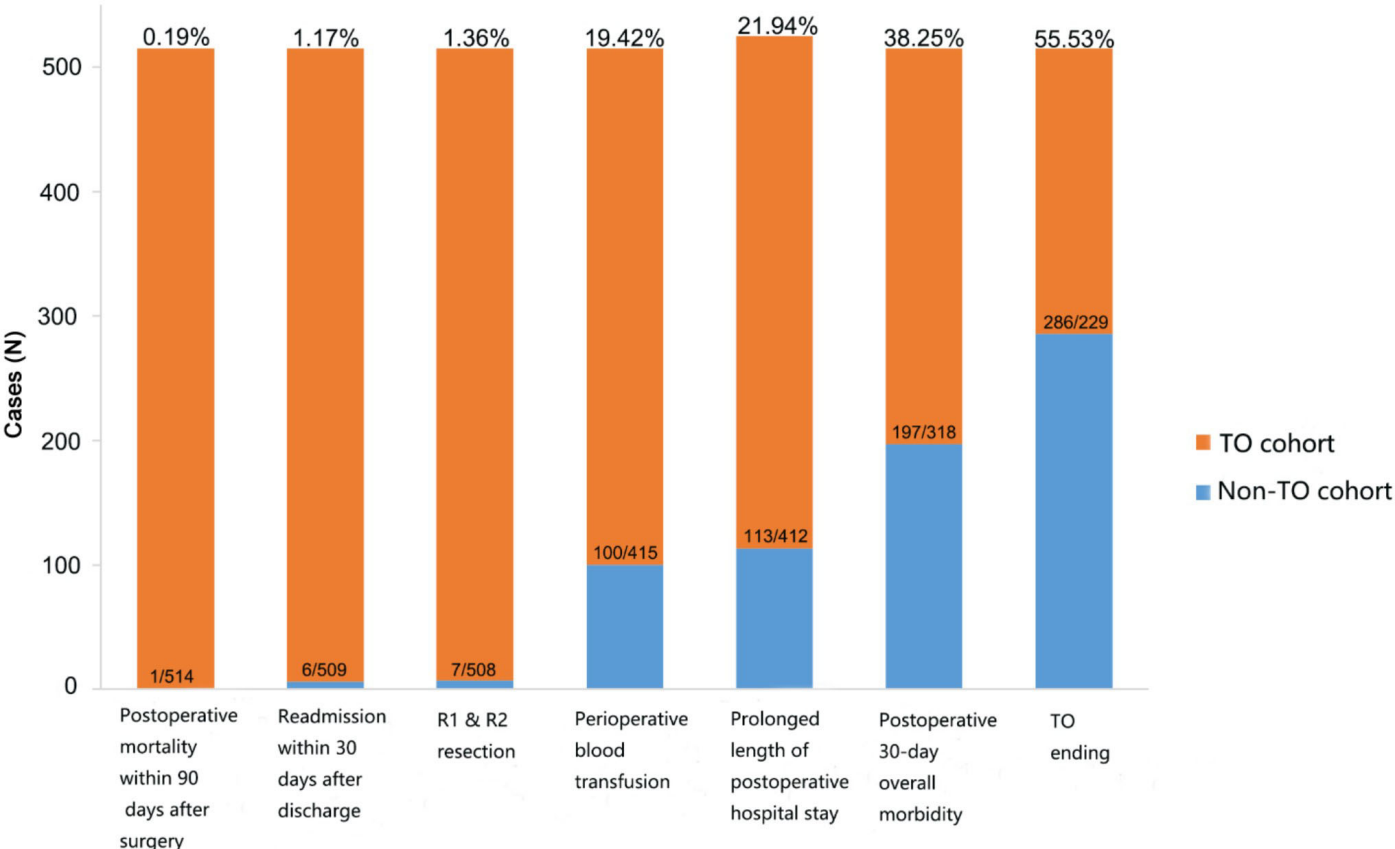
Distribution of outcome measures between the two groups.

Among 515 patients who underwent curative-intent LLR for HCC enrolled in the study, a total of 286 (55.5%) patients did not achieve TO, and 229 (44.5%) patients achieved TO. There was 1 (0.19%) patient who died within 90 days after surgery, 6 (1.17%) patients were readmitted within 30 days after discharge, 7 (1.36%) patients were subjected to R1 or R2 resection, 100 (19.42%) patients underwent perioperative blood transfusion, 113 (21.94%) patients had prolonged postoperative length of hospital stay, and 197 (38.25%) patients encountered postoperative 30-day morbidity (**Figure 1**).

### Baseline characteristics

There are 515 people in all queues (**Table 1**), which shows the comparison of the characteristics of the baseline between the TO group and the non-TO group. Compared with TO group patients, non-TO patients had a higher proportion of age > 70 years, obesity, portal hypertension, high ALBI grade, high NLR grade, AST level (>40 U/L), location of tumor (7/8 segment), largest tumor size (>5 cm), multiple tumors, macroscopic vascular invasion, intraoperative blood loss (>400 ml), and major hepatectomy (all *p* < 0.05).

**Table 1:** Comparisons of clinical characteristics among the two groups according to textbook outcomes.

	Overall cohort	TO cohort	Non-TO cohort	
VARIABLES	(*N* = 515)	(*N* = 229)	(*N* = 286)	*P*-VALUE
Age > 70 years	72 (14.0)	24 (10.5)	48 (16.8)	0.042
Male	420 (81.6)	193 (84.3)	227 (79.4)	0.171
Cigarette smoking	199 (38.6)	86 (37.6)	113 (39.5)	0.717
Alcohol drinking	153 (29.7)	68 (29.7)	85 (29.7)	1.000
Diabetes mellitus	67 (13.0)	23 (10.0)	44 (15.4)	0.087
Obesity	47 (9.1)	12 (5.2)	35 (12.2)	0.008
ASA score > 2	88 (17.1)	33 (14.4)	55 (19.2)	0.159
HBV (+)	425 (82.5)	192 (83.8)	233 (81.5)	0.560
Cirrhosis	376 (73.0)	165 (72.1)	211 (73.8)	0.735
Portal hypertension	152 (29.5)	50 (21.8)	102 (35.7)	0.001
Child–Pugh grade B	35 (6.8)	11 (4.8)	24 (8.4)	0.116
High ALBI grade	379 (73.6)	153 (66.8)	226 (79.0)	0.003
High NLR grade	124 (24.1)	38 (16.6)	86 (30.1)	<0.001
ALT > 40 IU/L	145 (28.2)	55 (24.0)	90 (31.5)	0.062
AST > 40 IU/L	146 (28.3)	51 (22.3)	95 (33.2)	0.006
PLT < 100 10^9^/L	129 (25.0)	49 (21.4)	80 (28.0)	0.108
AFP > 20 μg/L	276 (53.6)	124 (54.1)	152 (53.1)	0.891
Tumor in segment 7/8	150 (29.1)	50 (21.8)	100 (35.0)	0.002
Maximum tumor size > 5 cm	121 (23.5)	19 (8.3)	48 (16.8)	<0.001
Multiple tumors	67 (13.0)	34 (14.8)	87 (30.4)	0.005
Macroscopic vascular invasion	24 (4.7)	5 (2.2)	19 (6.6)	0.020
Intraoperative blood loss > 400 ml	104 (20.2)	13 (5.7)	91 (31.8)	<0.001
Major hepatectomy	64 (12.4)	12 (5.2)	52 (18.2)	<0.001
Non-anatomical hepatectomy	248 (48.2)	115 (50.2)	133 (46.5)	0.453

ASA, American Society of Anesthesiologists; HBV, hepatitis B virus; ALBI, albumin–bilirubin grade; AFP, alpha fetoprotein; NLR, neutrophil-to-lymphocyte ratio; ALT, alanine aminotransferase; AST, aspartate aminotransferase; PLT, platelets.

### Independent risk factors associated with non-TO

Univariate and multivariate logistic regression analysis of preoperative and postoperative variables confirmed several independent risk factors related to non-TO (**Table 2**). Variables with *p* < 0.1 were included in the multivariable logistic regression model. In the preoperative model, multiple regression analysis data showed that obesity, portal hypertension, high ALBI classification, high NLR classification, tumor in segment 7/8, maximum tumor size > 5 cm, and multiple tumors were identified as independent risk factors of non-TO. In addition, in the postoperative predictive model, age > 70 years, obesity, portal hypertension, high ALBI grade, high NLR grade, tumor in segment 7/8, intraoperative blood loss > 400 ml, and major hepatectomy were independent risk factors associated with a higher incidence of non-TO.

**Table 2:** Univariable and multivariable logistic regression analyses of risk factors associated with not achieving a textbook outcome following hepatectomy for HCC.

	Univariable analysis		Multivariable logistic regression analysis			
			Preoperative predictive model		Postoperative predictive model	
	Odds ratio (95% CI)	*p*	Odds ratio (95% CI)	*p*	Odds ratio (95% CI)	*p*
Preoperative variables						
Age > 70 years	1.723 (1.020–2.910)	0.042	NS	0.092	2.093 (1.187–3.690)	0.011
Male	1.393 (0.882–2.200)	0.155				
Cigarette smoking	1.061 (0.743–1.516)	0.743				
Alcohol drinking	1.001 (0.684–1.465)	0.995				
Diabetes mellitus	1.628 (0.951–2.787)	0.075	NS	0.188	NS	0.268
Obesity	2.522 (1.277–4.979)	0.008	2.137 (1.044–4.373)	0.038	2.284 (1.086–4.802)	0.029
ASA score > 2	1.414 (0.882–2.266)	0.150				
HBV (+)	0.847 (0.534–1.344)	0.481				
Cirrhosis	1.091 (0.738–1.613)	0.661				
Portal hypertension	1.985 (1.336–2.949)	0.001	1.859 (1.214–2.848)	0.004	1.854 (1.193–2.881)	0.006
High ALBI grade	1.871 (1.259–2.780)	0.002	1.638 (1.073–2.500)	0.022	1.567 (1.004–2.444)	0.048
High NLR grade	2.161 (1.406–3.323)	< 0.001	1.993 (1.261–3.151)	0.003	2.070 (1.294–3.310)	0.002
ALT > 40 IU/L	1.453 (0.981–2.151)	0.062	NS	0.128	NS	0.397
AST > 40 IU/L	1.736 (1.167–2.581)	0.006	NS	0.123	NS	0.134
PLT < 100 10^9^/L	1.427 (0.949–2.145)	0.088	NS	0.898	NS	0.795
AFP > 20 μg/L	0.961 (0.678–1.361)	0.821				
Tumor in segment 7/8	1.925 (1.294–2.862)	0.001	1.829 (1.200–2.788)	0.005	1.623 (1.041–2.532)	0.033
Maximum tumor size > 5 cm	2.507 (1.610–3.904)	< 0.001	2.318 (1.445–3.717)	< 0.001	NS	0.164
Multiple tumors	2.229 (1.270–3.913)	0.005	1.984 (1.097–3.588)	0.024	NS	0.086
Macroscopic vascular invasion	3.188 (1.172–8.675)	0.023	NS	0.129	NS	0.387
Intraoperative variables						
Blood loss > 400 ml	7.754 (4.202–14.31)	< 0.001			6.873 (3.588–12.82)	< 0.001
Major hepatectomy	4.019 (2.089–7.731)	< 0.001			3.461 (1.704–7.032)	0.001
Non-anatomical hepatectomy	1.160 (0.819–1.643)	0.402				

ASA, American Society of Anesthesiologists; HBV, hepatitis B virus; ALBI, albumin-bilirubin grade; AFP, alpha fetoprotein; NLR, neutrophil-to-lymphocyte ratio; ALT, alanine aminotransferase; AST, aspartate aminotransferase; PLT, Platelets. Ns, no significance.

### Preoperative and postoperative nomogram models for predicting non-TO

**Figure 2:**
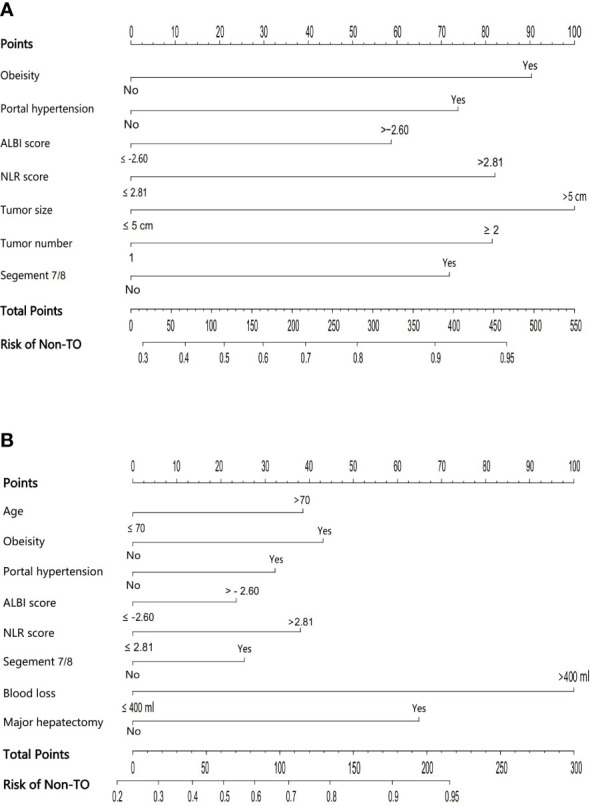
The preoperative **(A)** and postoperative **(B)** nomogram models for predicting non-TO.

**Figure 3:**
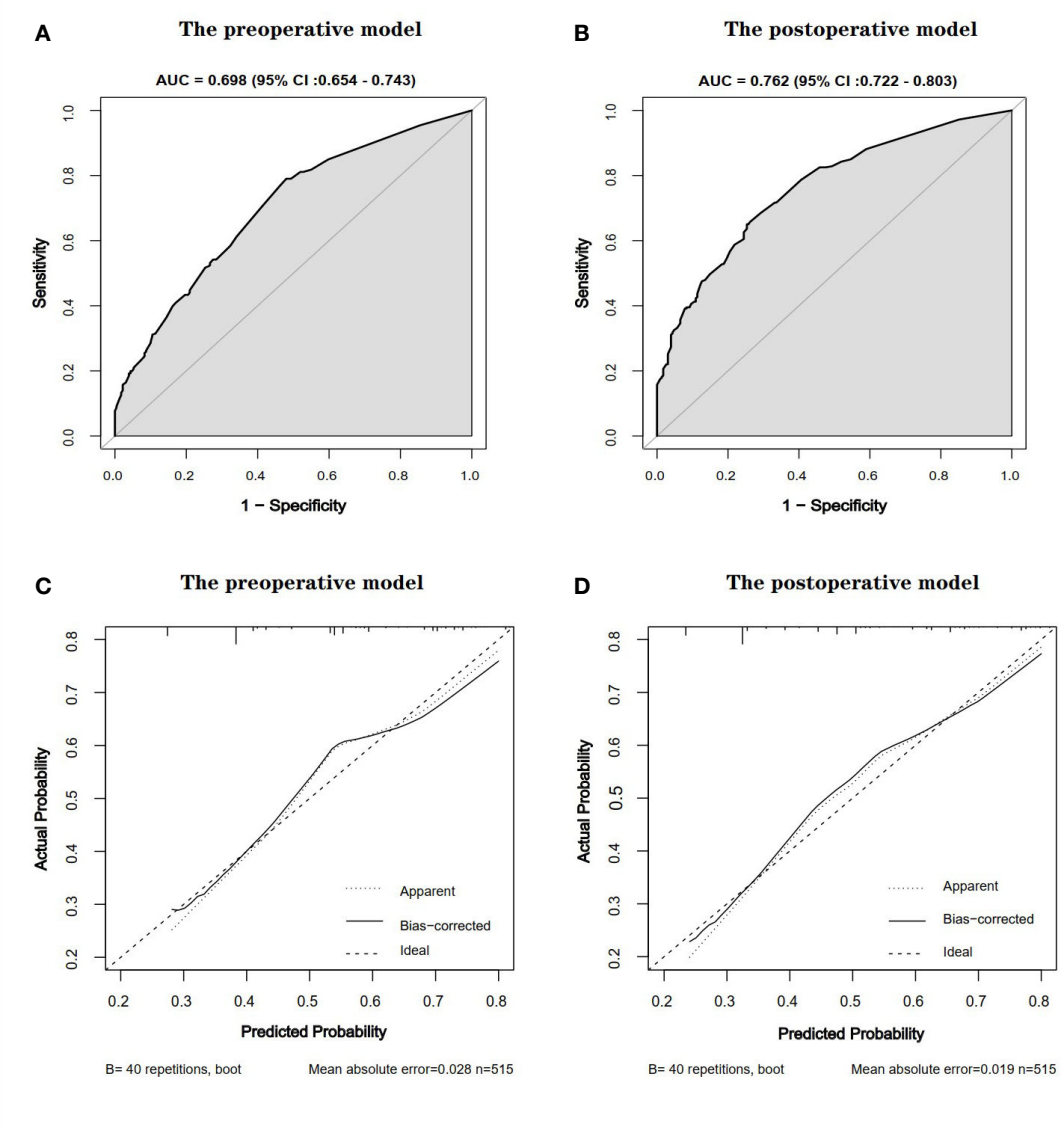
Receiver operating characteristic curves [**(A)**, preoperative model; **(C)**, postoperative model] and calibration charts [**(B)**, preoperative model; **(D)**, postoperative model] for predicting preoperative and postoperative non-TO models. The calibration chart compares preoperative and postoperative results with actual results. The dotted line is the reference line, indicating the position of the ideal nomogram. The solid line represents the bootstrap performance of 40 samples of the nomogram. When the predicted probability is plotted against the actual probability, the calibration plot is close to the dotted line, indicating that the calibration plot for the nomogram is good in both groups. AUC, area under the curve; CI, confidence interval.

Based on the results of multivariate logistic regression model, two nomogram models were established to predict non-TO before and after surgery. The preoperative predictive nomogram model included only preoperative variables, while the postoperative predictive nomogram model included preoperative and intraoperative variables. As shown in **Figure 2**, each predictor has a specific score on the corresponding point line. By adding each risk factor score to get a total score, a vertical line can be drawn down from that particular point to obtain the probability of non-TO. The receiver operating characteristic curves of the two models are demonstrated in **Figures 3A, C** (AUC = 0.698; 95% CI: 0.654–0.743 vs. AUC = 0.722; 95% CI: 0.722–0.803). Meanwhile, **Figures 3B, D** show that the calibration plots of preoperative and postoperative nomograms had acceptable fit and consistency between the predictive value and actual observation.

### Predictive accuracy of two nomogram models for non-TO

**Figure 4:**
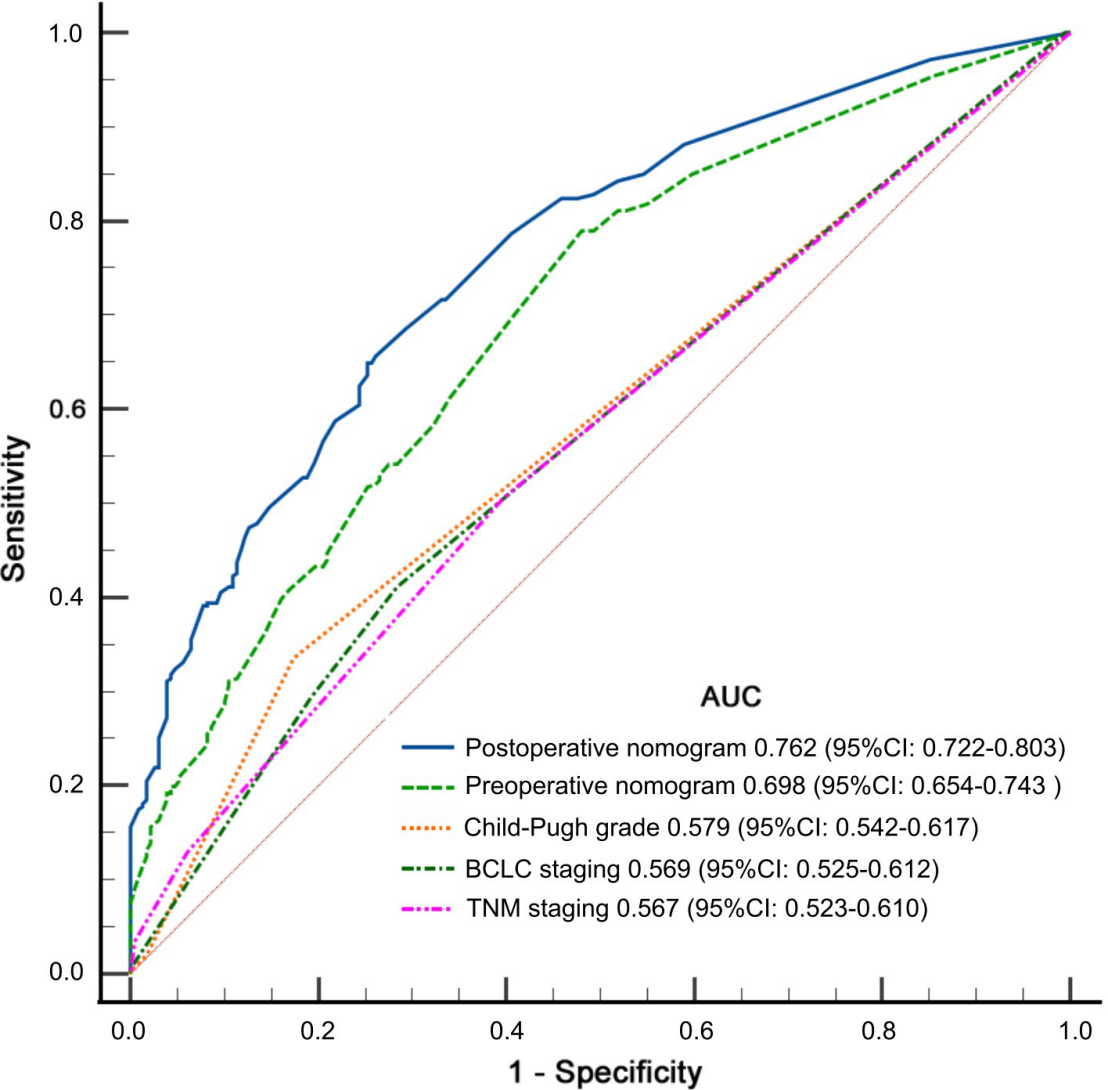
Receiver operating characteristic curves of preoperative and postoperative predictive models, and Child–Pugh grade, tumor-node-metastasis (8th TNM) staging, and Barcelona Clinic Liver Cancer (BCLC) staging for predicting non-textbook outcomes (non-TO).

Using the ROC curves, the predictive power of index was evaluated. The comparisons of the discriminatory ability of the two predictive models, Child–Pugh grade, 8th TNM staging, and BCLC staging for predicting the non-TO are shown in **Figure 4**. The AUCs of the preoperative and postoperative nomogram models were (0.698; 0.654–0.743) and (0.762; 0.722–0.803), respectively, which were superior to those of Child–Pugh grade (0.579; 0.542–0.617), BCLC staging (0.569; 0.525–0.612), and 8th TNM staging (0.567; 0.523–0.610).

## Discussion

TO can be used not only as a comprehensive index of the quality of surgical treatment, but also as an unalterable predictor of long-term recovery of many malignant tumors. Therefore, since some kinds of influencing factors are likely to be improved or reduced, it is particularly important to clarify the correlation between clinical medical variables and non-TO after tumor surgery. The present study develops preoperative and postoperative nomograms. To our knowledge, this is the first study to predict non-TO after LLR for HCC.

Among 515 patients who underwent LLR for HCC, 229 patients (44.5%) achieved TO in the whole cohort, which was better than previous studies (33.3%–34.4%) (24, 25). The predictors we mainly analyzed include the general condition of the patients, liver function, immune inflammation of the body, tumor burden, tumor location, and surgical procedure. The preoperative prediction model integrates portal hypertension, high ALBI grade, high NLR grade, tumor site, maximum tumor size > 5 cm, and multiple tumors. The discriminatory ability of the preoperative nomogram nearly reached 0.7. Previous studies had found that preoperative low and high BMI were associated with lower chances of achieving TO (25). Similarly, obesity, BMI > 28 kg/m^2^, also had a negative impact on TO in the present study. High ALBI and portal hypertension, indicators of poor liver function, were proved in previous studies (14, 17, 25). Insufficient liver reserve is closely related to postoperative complications, including liver failure and massive ascites. As an easily calculated and inexpensive marker, preoperative NLR tended to reflect the system inflammation of the human body and long-term prognosis of several malignancies. Owing to chronic infection with HBV or hepatitis C virus (HCV), patients with HCC and high NLR grade have neutrophilic leukocytosis and lymphocytopenia, which demonstrated that the balance is tilted towards tumor inflammatory response, leading to a disappointing surgical outcome. Previous studies have shown that NLR is independently associated with postoperative complications and in-hospital mortality (26, 27). In our present study, similarly, we also confirmed that NLR is an independent risk factor for TO, suggesting the potential usefulness of deducing elevated NLR before LLR.

Different from traditional open hepatectomy, LLR has the characteristic of magnifying the surgical field, while a limited operating space and the lack of actual touch mean higher surgical difficulty and a longer learning curve for hepatobiliary surgeons. Tumor location as well as tumor burden including tumor size and number would directly affect the complexity of LLR. In the study, tumor size > 5 cm, multiple tumors, and segment 7/8 were predictors for TO by using logistic regression analysis. For lesions located at segment 7/8, the occurrence of postoperative complications was significantly higher than other segments, because of the great difficulty level of tumor location (28). The feasibility and safety of LLR for tumor size ≤ 5 cm were widely recognized by surgeons (29). With the progress and development of minimally invasive surgery, large (>5 cm) and even giant (>10 cm) malignant liver tumors are not a contraindication for LLR (30–32). In our study, however, a tumor size greater than 5 cm made it more difficult to achieve TO. In the postoperative nomogram, we can find that major hepatectomy, instead of tumor size and number, was a risk factor for TO. It is believed that the greater the tumor burden, the greater the extent of liver resection. After multivariate logistic regression adjustment, the authentic postoperative independent predictor depends on major hepatectomy rather than tumor burden for patients with HCC who were subjected to LLR. In addition, the treatment for large and giant HCC using LLR is difficult and requires the operator’s proficient minimally invasive technique. Whether traditional open liver resection is more conducive to achieving TO deserves further study.

The main limitation of the paper mainly arises from its retrospective nature, rendering it susceptible to selection bias. Second, this study included patients with HCC who received laparoscopic hepatectomy. Therefore, further assessment is required to implement whether HCC patients treated with open hepatectomy could be used as reference. Third, the patients in this study also received treatment in China, and most HCC patients have a background of HBV infection. However, in Europe and the United States, HCV infection and excessive drinking are the risk factors (33, 34). The predictive models need an external validation cohort to improve the model reliability. In addition, prospective studies are needed to further confirm the reliability of nomograms. Fourth, this study focused on primary HCC, and recurrent HCC needs further research in the future.

In conclusion, the present study systematically revealed the factors influencing the non-TO of LLR for HCC patients. In addition, two nomograms were conducted for predicting non-TO, which were superior to the Child–Pugh grade, TNM staging, and BCLC staging and could help surgeons make individualized treatment plans for HCC patients to achieve TO.

## Data availability statement

The raw data supporting the conclusions of this article will be made available by the authors, without undue reservation.

## Author contributions

G-LX, LL, T-WY, and F-QX contributed equally to this work. C-WZ and J-WL had full access to all the data in the study and take responsibility for the integrity of the data and the accuracy of the data analysis. Study concept and design: G-LX, C-WZ, and J-WL. Acquisition, analysis, or interpretation of data: LL, T-WY, F-QX, D-DW, Y-MX, K-JZ, T-WF, and W-FY. Drafting of the manuscript: G-LX and LL. Critical revision of the manuscript for important intellectual content: W-FY, J-WL, and C-WZ. Statistical analysis: LL, T-WY, and F-QX. Obtained funding: C-WZ, D-DW, and Y-MX. Administrative, technical, or material support: C-WZ, J-WL, and W-FY. Study supervision: C-WZ and J-WL. All authors contributed to the article and approved the submitted version.

## References

[1] McGlynn KA Petrick JL El-Serag HB. Epidemiology of hepatocellular carcinoma. Hepatol (Baltimore Md) (2021) 73 Suppl 1(Suppl 1):4–13. doi: 10.1002/hep.31288 PMC7577946 32319693

[2] Llovet JM Kelley RK Villanueva A Singal AG Pikarsky E Roayaie S. Hepatocellular carcinoma. Nat Rev Dis primers (2021) 7(1):6. doi: 10.1038/s41572-020-00240-3 33479224 PMC13537433

[3] Makino Y Yamanoi A Kimoto T El-Assal ON Kohno H Nagasue N. The influence of perioperative blood transfusion on intrahepatic recurrence after curative resection of hepatocellular carcinoma. Am J gastroenterol (2000) 95(5):1294–300. doi: 10.1111/j.1572-0241.2000.02028.x 10811342

[4] Yamamoto J Kosuge T Takayama T Shimada K Yamasaki S Ozaki H. Perioperative blood transfusion promotes recurrence of hepatocellular carcinoma after hepatectomy. Surgery (1994) 115(3):303–9. 8128355

[5] Liu L Wang Z Jiang S Shao B Liu J Zhang S. Perioperative allogenenic blood transfusion is associated with worse clinical outcomes for hepatocellular carcinoma: a meta-analysis. PloS One (2013) 8(5):e64261. doi: 10.1371/journal.pone.0064261 23741309 PMC3669337

[6] Kong J Li G Chai J Yu G Liu Y Liu J. Impact of postoperative complications on long-term survival after resection of hepatocellular carcinoma: A systematic review and meta-analysis. Ann Surg Oncol (2021) 28(13):8221–33. doi: 10.1245/s10434-021-10317-2 34160708

[7] Kabir T Syn NL Tan ZZX Tan HJ Yen C Koh YX. Predictors of post-operative complications after surgical resection of hepatocellular carcinoma and their prognostic effects on outcome and survival: A propensity-score matched and structural equation modelling study. Eur J Surg Oncol (2020) 46(9):1756–65. doi: 10.1016/j.ejso.2020.03.219 32345496

[8] van der Kaaij RT de Rooij MV van Coevorden F Voncken FEM Snaebjornsson P Boot H. Using textbook outcome as a measure of quality of care in oesophagogastric cancer surgery. Br J surgery. (2018) 105(5):561–9. doi: 10.1002/bjs.10729 29465746

[9] Kulshrestha S Bunn C Patel PM Sweigert PJ Eguia E Pawlik TM. Textbook oncologic outcome is associated with increased overall survival after esophagectomy. Surgery (2020) 168(5):953–61. doi: 10.1016/j.surg.2020.05.038 32675034

[10] Kalff MC Vesseur I Eshuis WJ Heineman DJ Daams F van der Peet DL. The association of textbook outcome and long-term survival after esophagectomy for esophageal cancer. Ann Thorac surgery. (2021) 112(4):1134–41. doi: 10.1016/j.athoracsur.2020.09.035 33221197

[11] Xu SJ Lin LQ Chen C Chen TY You CX Chen RQ. Textbook outcome after minimally invasive esophagectomy is an important prognostic indicator for predicting long-term oncological outcomes with locally advanced esophageal squamous cell carcinoma. Ann Trans Med (2022) 10(4):161. doi: 10.21037/atm-22-506 PMC8908120 35280418

[12] Yang CC Tian YF Liu WS Chou CL Cheng LC Chu SS. The association between the composite quality measure "textbook outcome" and long term survival in operated colon cancer. Medicine (2020) 99(40):e22447. doi: 10.1097/MD.0000000000022447 33019430 PMC7535643

[13] Kulshrestha S Vigneswaran WT Pawlik TM Baker MS Luchette FA Raad W. Assessment of textbook outcome after surgery for stage I/II non-small cell lung cancer. Semin Thorac Cardiovasc Surg (2022) 34(4):1351–9. doi: 10.1053/j.semtcvs.2021.08.009 PMC8848000 34411699

[14] Tsilimigras DI Sahara K Moris D Mehta R Paredes AZ Ratti F. Assessing textbook outcomes following liver surgery for primary liver cancer over a 12-year time period at major hepatobiliary centers. Ann Surg Oncol (2020) 27(9):3318–27. doi: 10.1245/s10434-020-08548-w 32388742

[15] D'Silva M Cho JY Han HS Yoon YS Lee HW Lee JS. Association between achieving textbook outcomes and better survival after laparoscopic liver resection in the anterolateral segments in patients with hepatocellular carcinoma. J hepato-biliary-pancreatic Sci (2022) 29(8):855–62. doi: 10.1002/jhbp.1148 35389551

[16] Lazarides AL Cerullo M Moris D Brigman BE Blazer DG Eward WC. Defining a textbook surgical outcome for patients undergoing surgical resection of intermediate and high-grade soft tissue sarcomas of the extremities. J Surg Oncol (2020) 122(5):884–96. doi: 10.1002/jso.26087 32691847

[17] Xu FQ Ye TW Wang DD Xie YM Zhang KJ Cheng J. Association of preoperative albumin-bilirubin with surgical textbook outcomes following laparoscopic hepatectomy for hepatocellular carcinoma. Front Oncol (2022) 12:964614. doi: 10.3389/fonc.2022.964614 35965571 PMC9373871

[18] Johnson PJ Berhane S Kagebayashi C Satomura S Teng M Reeves HL. Assessment of liver function in patients with hepatocellular carcinoma: a new evidence-based approach-the ALBI grade. J Clin Oncol (2015) 33(6):550–8. doi: 10.1200/JCO.2014.57.9151 PMC4322258 25512453

[19] Mano Y Shirabe K Yamashita Y Harimoto N Tsujita E Takeishi K. Preoperative neutrophil-to-lymphocyte ratio is a predictor of survival after hepatectomy for hepatocellular carcinoma: a retrospective analysis. Ann surgery. (2013) 258(2):301–5. doi: 10.1097/SLA.0b013e318297ad6b 23774313

[20] Strasberg SM Phillips C. Use and dissemination of the brisbane 2000 nomenclature of liver anatomy and resections. Ann surgery. (2013) 257(3):377–82. doi: 10.1097/SLA.0b013e31825a01f6 22895397

[21] Kim Y Gani F Lucas DJ Ejaz A Spolverato G Canner JK. Early versus late readmission after surgery among patients with employer-provided health insurance. Ann surgery. (2015) 262(3):502–11; discussion 9-11. doi: 10.1097/SLA.0000000000001429 26258319

[22] Hermanek P Wittekind C. The pathologist and the residual tumor (R) classification. Pathol Res practice (1994) 190(2):115–23. doi: 10.1016/S0344-0338(11)80700-4 8058567

[23] Youden WJ. Index for rating diagnostic tests. Cancer (1950) 3(1):32–5. doi: 10.1002/1097-0142(1950)3:1<32::AID-CNCR2820030106>3.0.CO;2-3 15405679

[24] Merath K Chen Q Bagante F Beal E Akgul O Dillhoff M. Textbook outcomes among Medicare patients undergoing hepatopancreatic surgery. Ann surgery. (2020) 271(6):1116–23. doi: 10.1097/SLA.0000000000003105 30499800

[25] Liu ZP Yao LQ Diao YK Chen ZX Feng ZH Gu WM. Association of preoperative body mass index with surgical textbook outcomes following hepatectomy for hepatocellular carcinoma: A multicenter study of 1206 patients. Ann Surg Oncol (2022). doi: 10.1245/s10434-022-11721-y 35419755

[26] Wu HL Liu HY Liu WC Hou MC Tai YH. A predictive model incorporating inflammation markers for high-grade surgical complications following liver resection for hepatocellular carcinoma. J Chin Med Assoc JCMA (2022) 85(8):845–52. doi: 10.1097/JCMA.0000000000000713 PMC12755644 35316229

[27] Mahassadi AK Anzouan-Kacou Kissi H Attia AK. The prognostic values of neutrophil-to-lymphocyte ratio and platelet-to-Lymphocyte ratio at baseline in predicting the in-hospital mortality in black African patients with advanced hepatocellular carcinoma in palliative treatment: A comparative cohort study. Hepatic Med evidence Res (2021) 13:123–34. doi: 10.2147/HMER.S333980 PMC8686837 34938131

[28] Gau RY Yu MC Tsai HI Lee CH Kuo T Lee KC. Laparoscopic liver resection should be a standard procedure for hepatocellular carcinoma with low or intermediate difficulty. J personalized Med (2021) 11(4):266. doi: 10.3390/jpm11040266 PMC8067022 33918197

[29] Buell JF Cherqui D Geller DA O'Rourke N Iannitti D Dagher I. The international position on laparoscopic liver surgery: The Louisville statement, 2008. Ann surgery. (2009) 250(5):825–30. doi: 10.1097/SLA.0b013e3181b3b2d8 19916210

[30] Shelat VG Cipriani F Basseres T Armstrong TH Takhar AS Pearce NW. Pure laparoscopic liver resection for large malignant tumors: does size matter? Ann Surg Oncol (2015) 22(4):1288–93. doi: 10.1245/s10434-014-4107-6 25256130

[31] Ai JH Li JW Chen J Bie P Wang SG Zheng SG. Feasibility and safety of laparoscopic liver resection for hepatocellular carcinoma with a tumor size of 5-10 cm. PloS One (2013) 8(8):e72328. doi: 10.1371/journal.pone.0072328 23991092 PMC3749106

[32] Fang Q Xie QS Chen JM Shan SL Xie K Geng XP. Long-term outcomes after hepatectomy of huge hepatocellular carcinoma: A single-center experience in China. Hepatobiliary pancreatic Dis Int HBPD Int (2019) 18(6):532–7. doi: 10.1016/j.hbpd.2019.09.001 31543313

[33] Estes C Razavi H Loomba R Younossi Z Sanyal AJ. Modeling the epidemic of nonalcoholic fatty liver disease demonstrates an exponential increase in burden of disease. Hepatology (2018) 67(1):123–33. doi: 10.1002/hep.29466 PMC5767767 28802062

[34] Bertuccio P Turati F Carioli G Rodriguez T La Vecchia C Malvezzi M. Global trends and predictions in hepatocellular carcinoma mortality. J Hepatol (2017) 67(2):302–9. doi: 10.1016/j.jhep.2017.03.011 28336466

